# miR‐126‐5p enhances radiosensitivity of lung adenocarcinoma cells by inhibiting EZH2 via the KLF2/BIRC axis

**DOI:** 10.1111/jcmm.17135

**Published:** 2022-03-24

**Authors:** Fushi Han, Dongdong Huang, Jinqian Meng, Jiapeng Chu, Meng Wang, Shuzhen Chen

**Affiliations:** ^1^ Department of Nuclear Medicine Tongji Hospital Tongji University School of Medicine Shanghai China; ^2^ Department of Emergency Medicine Shanghai Pulmonary Hospital Tongji University School of Medicine Shanghai China; ^3^ Department of Radiology Tongji Hospital Tongji University School of Medicine Shanghai China; ^4^ Department of Cardiology Tongji Hospital Tongji University School of Medicine Shanghai China; ^5^ Department of Radiotherapy Tongji Hospital Tongji University School of Medicine Shanghai China

**Keywords:** BIRC, EZH2, KLF2, lung adenocarcinoma, miR‐126‐5p, radiosensitivity

## Abstract

Radiotherapy is a common method for the treatment of lung adenocarcinoma, but it often fails due to the relative non‐susceptibility of lung adenocarcinoma cells to radiation. We aimed to discuss the related mechanisms by which miR‐126‐5p might mediate radiosensitivity of lung adenocarcinoma cells. The binding affinity between miR‐126‐5p and EZH2 and between KLF2 and BIRC5 was identified using multiple assays. A549 and H1650 cells treated with X‐ray were transfected with miR‐126‐5p mimic/inhibitor, oe‐EZH2, or si‐KLF2 to detect cell biological functions and radiosensitivity. Finally, lung adenocarcinoma nude mouse models were established. miR‐126‐5p and KLF2 were poorly expressed, while EZH2 and BIRC5 were upregulated in lung adenocarcinoma tissues and cells. miR‐126‐5p targeted EZH2 to promote the KLF2 expression so as to inhibit BIRC5 activation. Both in vitro and in vivo experiments verified that elevated miR‐126‐5p inhibited cell migration and promoted apoptosis to enhance the sensitivity of lung adenocarcinoma cells to radiotherapy via the EZH2/KLF2/BIRC5 axis. Collectively, miR‐126‐5p downregulated EZH2 to facilitate the sensitivity of lung adenocarcinoma cells to radiotherapy via KLF2/BIRC5.

## 1 INTRODUCTION

As a major component of lung cancer, lung adenocarcinoma occupies about 40% of all patients with lung cancer.^1^ The development of lung adenocarcinoma is affected by multiple factors, such as smoking, environment toxins as well as genetic mutations, leading to the tumourigenesis.^2^ In recent years, chemotherapy and radiotherapy are regarded as the effective therapies to improve the survival rate and prognosis of lung adenocarcinoma sufferers.^3^ Unfortunately, many patients with lung adenocarcinoma show an increased resistance to radiotherapy.^4^ Therefore, it is of great importance to understand the potential mechanisms underlying radioresistant lung adenocarcinoma.

Accumulating evidences have proved that aberrant expression of microRNAs (miRNAs) is involved in the multiple human cancers, including lung adenocarcinoma, suggesting that they function as the promising biomarkers for lung adenocarcinoma.^5^, ^6^ miR‐126‐5p, as an intronic miRNA, acts as a tumour suppressor in multiple malignancies, including non‐small cell lung cancer (NSCLC).^7^ A prior study has exhibited that a variety of miRNAs, including miR‐126‐3p, is implicated in the progression of lung adenocarcinoma.^8^ In addition, miRNAs possess the potential to enhance the radiotherapy efficacy for lung adenocarcinoma.^9^ However, little information concerning the impact of miR‐126‐5p on the radiosensitivity of lung adenocarcinoma cells. Moreover, the bioinformatics analysis in the current study predicted that miR‐126‐5p might target enhancer of zeste homolog 2 (EZH2). EZH2 is capable of affecting the occurrence and progression of human cancers by modulation of cell biological processes.^10^ Existing literature has reported that EZH2 is dysregulated in lung adenocarcinoma.^11^ The roles of EZH2 in prostate cancer cell resistance to radiotherapy have been explored.^12^ However, the mechanism by which miR‐126‐5p affects radiosensitivity of lung adenocarcinoma cells by targeting EZH2 is still poorly understood. Hence, we hypothesised that the miR‐126‐5p‐mediated EZH2 might alter radiosensitivity of lung adenocarcinoma cells.

## 2 METHODS

### 2.1 Ethic statement

This study was performed with the approval of the Ethics Committee of Shanghai Pulmonary Hospital affiliated to Tongji University School of Medicine. This study was carried out in strict accordance with the *Declaration of Helsinki* and all enrolled patients were aware of the full details regarding the study and experiments with informed consent form signed. All animal experiments were approved by the Ethics Review Committee for Shanghai Pulmonary Hospital affiliated to Tongji University School of Medicine. Animal experiments were approved according to the guidelines of the Care and Use of Laboratory Animals by the National Institute of Health.

### 2.2 Bioinformatics analysis

Lung adenocarcinoma‐related miRNA expression dataset GSE135918 was downloaded through the Gene Expression Omnibus database. The dataset contains five adjacent normal tissues (normal) and five lung adenocarcinoma tissues. The R language ‘limma’ package was used for differential analysis of the gene expression, with |logFoldChange| >2, and *p* < 0.01 as the screening criteria. Gene Expression Profiling Interactive Analysis 2 (GEPIA2) tool was used for differential analysis of the gene expression of lung adenocarcinoma tissues in the The Cancer Genome Atlas database compared to normal samples. The bioinformatics target gene prediction tool StarBase was used to predict the target genes of miR‐126‐5p, and the lung adenocarcinoma‐related genes were searched through the GeneCards database. The Venn tool was employed to identify the overlap between target genes and disease‐related genes. In order to further screen target genes, the Coexpedia website was utilised to analyse the co‐expression relationship network between candidate target genes, and to determine candidate genes based on the co‐expression relationship score. The binding site of miR‐126‐5p and target gene was obtained through StarBase website. Next, the downstream regulatory factors of the target gene were predicted. The interacting proteins were searched in the GeneCards database, and the jvenn tool was used to find the overlap between the interacting proteins and the significantly downregulated differential genes in lung adenocarcinoma. Similarly, the STRING website was employed to analyse the interaction network among genes, and the Cytoscape 3.5.1 software was employed to visualise the interaction network. The GEPIA2 tool was used to further analyse the correlation among genes, and the JASPAR website was utilised to predict the possible binding sites of transcription factors in gene promoter regions.

### 2.3 Study patients

A total of 78 pairs of lung adenocarcinoma tissues and adjacent normal tissues (2 cm away from the tumour) were obtained from the patients with lung adenocarcinoma who underwent surgical treatment in thoracic surgery in Shanghai Pulmonary Hospital affiliated to Tongji University School of Medicine from September 2015 to June 2017. The frozen tissues were stored in liquid nitrogen. The other part of the tissues was embedded in paraffin for the following experiments. All patients were diagnosed by histopathology and had not undergone any anticancer treatment before the surgical separation of the tissues. The clinical sample information is shown in Table S1. With the telephone follow‐up and outpatient information inquiry, the physical conditions of postoperative patients at different periods were reviewed. This follow‐up started from the diagnosis of lung adenocarcinoma until death, and the follow‐up period was 2–30 months. The Kaplan‐Meier method was employed to analyse the survival rate of the patient.

### 2.4 In Situ Hybridisation (ISH)

ISH assay was implemented according to the manufacturer instructions of miRNA ISH miR‐126‐5p optimisation Kit (Exiqon, LNA™).^13^ The samples were scanned and photographed with the help of digital pathological scanning equipment (P250 FLASH, 3DHISTECH) and analysed by Image Pro Plus (version 5, Media Cybernetics). The positive rate was calculated as the integrated optical density/sum of product.

### 2.5 Immunohistochemistry

The separated tissues were deparaffinized, incubated with 3% H_2_O_2_ for 15 min to remove endogenous peroxidase, and subjected to antigen retrieval. Afterwards, the samples were blocked with 5% normal goat serum for 15 min and incubated with the following antibodies, rabbit polyclonal antibody against EZH2 (ab186006, 1: 100, Abcam), rabbit polyclonal antibody against KLF2 (PA5‐40591, 1: 100, Invitrogen), or rabbit polyclonal antibody against BIRC5 (10508–1‐AP, 1: 200, Proteintech Group Inc.) at 4°C overnight. Then, the tissues were added with biotinylated goat antirabbit immunoglobulin G (IgG, 1:1000, ab6721, Abcam) and incubated with horseradish peroxidase (HRP)‐streptomycin for 15 min. After treatment with diaminobenzidine solution (Beijing ZSGB Biotechnology Co., Ltd.) for 3–5 min, the tissues were counterstained with haematoxylin for 1–3 min, dehydrated, cleared, and sealed with neutral balsam. Finally, the Primo Star digital microscope (Motic China Group Co., Ltd.) was selected for observation. A yellow nucleus was considered to be positive.

### 2.6 Cell culture

Lung adenocarcinoma cell lines H1975, A549 and H1650, and human embryonic lung fibroblast cell line MRC‐5 were purchased from Shanghai Institute of Biological Sciences, Chinese Academy of Sciences (Shanghai, China). Lung adenocarcinoma cell line PC9 (National Infrastructure of Cell Line Resource) and H1573 (ATCC) were cultured in Dulbecco's Modified Eagle Medium (DMEM, Sigma‐Aldrich) containing 10% foetal bovine serum (FBS, HyClone). H1975 and H1650 were cultured in Roswell Park Memorial Institute‐1640 medium (Sigma‐Aldrich) containing 10% FBS. A549 cells were cultured in F‐12K medium (Sigma‐Aldrich) containing 10% FBS and placed in a humidified incubator with 5% CO_2_ at 37°C. All culture medium was added with penicillin and streptomycin to make the final concentration of 100 U/ml. Upon reaching 90% confluency, cells were sub‐cultured with 0.25% trypsin (T1300, Solarbio Science & Technology Co., Ltd.) at a ratio of 1:3. Two lung adenocarcinoma cell lines with relatively high and low expression of miR‐126‐5p were selected in subsequent experiments.

### 2.7 Radiotherapy treatment

The cells were irradiated with X‐Ray with the help of the Astrophysics Torrex X‐ray Inspection System (Model 120D, Scanray Corporation) at 115 kVp and 5 mA. The cells (1 × 10^5^ cells/ml) in the logarithmic growth phase were inoculated in DMEM containing 10% FBS and 100 U/ml penicillin‐streptomycin in an incubator at 37°C with 5% CO_2_. Irradiation was performed 24 h after transfection if needed. Cells were irradiated with X‐Ray of 10 Gy (dose rate: 0.341 Gy/min; radiation area: 10 × 10 cm^2^), and further cultured for 24 h for subsequent experiments.

### 2.8 Cell transduction

The cells were transduced with NC mimic, miR‐126‐5p mimic, NC inhibitor, miR‐126‐5p inhibitor, NC for gene overexpression (oe‐NC), overexpressed EZH2 (oe‐EZH2), mock (NC), KLF2, NC for small interfering RNA (siRNA) (si‐NC), siRNA against KLF2 (si‐KLF2), miR‐126‐5p mimic +si‐NC, and miR‐126‐5p mimic +si‐KLF2. The mimic, inhibitor, and transduced plasmids were purchased and synthesised in Genechem Co., Ltd. The day before transfection, the cells in the logarithmic growth phase were seeded on a 12‐well plate (1 × 10^5^ cells/ml). Upon reaching 70%–80% confluency, 800 μl serum‐free medium was added. The above‐mentioned plasmid mixed with lipo2000 (11668027, Thermo Fisher Scientific) were added to a 12‐well plate. The cells were cultured for 6 h and the medium was renewed for another 48‐h culturing.

### 2.9 Flow cytometry

Referring to the Annexin V‐Fluorescein Isothiocyanate (FITC) apoptosis kit (K201‐100, BioVisionMilpitas), cell apoptosis was analysed using FACScan Flow cytometer (Becton Dickinson and Company).

### 2.10 Immunofluorescence staining

The cells were fixed with PBS containing 4% paraformaldehyde, and then permeabilised with PBS containing 0.1% Triton X‐100 (Solarbio) for 20 min. Then, cells were blocked with PBS containing 5% bovine serum albumin (Amresco) for 1 h and incubated with the primary antibody against histone family member X (H2AX) phosphorylated on Ser 139 (γ‐H2AX) at 4°C overnight. Afterwards, the cells were incubated with Alexa Fluor‐488 labelled secondary antibody (1:300, Invitrogen, Thermo Fisher) for 60 min. The nucleus was stained with 4’,6‐diamidino‐2‐phenylindole. A fluorescence microscope (Eclipse E600, Nikon) was used for observation.

### 2.11 Transwell assay

Transwell migration assay was implemented based on previous method^14^ with basement membrane matrix (50 mg/L, Sigma‐Aldrich). The images were photographed under an inverted microscope (CKX41SF, Olympus).

### 2.12 RT‐qPCR

For the detection of genes EZH2, KLF2 and BIRC5, RNAiso plus (1 ml, Takara) was used for total RNA extraction. Nanodrop 2000 (Thermo Fisher) was utilised to detect the concentration of mRNA. RT of mRNA and cDNA synthesis was conducted using PrimeScript Strand cDNA synthesis kit (Takara). With GAPDH serving as an internal reference, RT‐qPCR was performed with SYBR Premix Ex Taq II (Takara) to evaluate the mRNA level. For the detection of miR‐126‐5p, miRNeasy mini kit (Cat. 217, 004, Qiagen) was employed to extract total miRNAs. RT was conducted in line with the instructions of TaqMan MiRNA (Applied Biosystems) RT kit. U6 acted as an internal reference, and TaqMan miRNA assays (Applied Biosystems) were used for miR‐126‐5p quantification. All RT‐qPCR analysis was implemented using Applied Biosystems 7900HT Fast Real‐Time PCR System (Applied Biosystems). The 2^−ΔΔCt^ method was used to quantify relative expression levels of target genes (Table S2).

### 2.13 Western blot analysis

Western blot analysis was implemented using the primary antibodies, rabbit anti‐EZH2 (ab186006, 1:2500, Abcam), rabbit antitrimethylation of H3K27 (H3K27me3) (ab272165, 1:1000, Abcam), rabbit anti‐KLF2 (PA5‐40591, 1:1000, Invitrogen) and rabbit anti‐BIRC5 (10508–1‐AP, 1:1000, Proteintech) as well as the secondary antibody working solution, HRP‐labelled goat antirabbit IgG (ab6721, 1:20 000, Abcam) or goat antimouse IgG (ab6789, 1:20,000, Abcam).^14^ Mouse anti‐β‐actin (ab8226, 1:5000, Abcam) served as the internal reference.

### 2.14 Dual‐luciferase reporter gene assay

The bioinformatics target gene prediction tool StarBase (http://starbase.sysu.edu.cn/) was used to predict the target gene of miR‐126‐5p, among which EZH2 was highly expressed in lung adenocarcinoma. The 3′untranslated region (3′UTR) of EZH2 was amplified from human genomic DNA and inserted into pmiR‐RB‐REPORT™ (Guangzhou RiboBio Co., Ltd.) by restriction enzyme digestion and ligation. Similarly, the 3′UTR mutant fragment of EZH2 was inserted into the pmiR‐RB‐REPORT™ control vector. Lung adenocarcinoma cells were transduced with miR‐126‐5p mimic and wild‐type (WT) reporter plasmid, miR‐126‐5p mimic and mutant‐type (MUT) reporter plasmid, NC mimic and WT reporter plasmid, and NC mimic and MUT reporter plasmid. The possible binding site of KLF2 on the BIRC5 promoter was predicted by the JASPAR database. The BIRC5 promoter and its corresponding mutant sequence were, respectively, constructed on the luciferase reporter vector pGL3 (Promega). The constructed vectors or Renilla pRLTK plasmid (Promega) were co‐transfected with KLF2 overexpression plasmid (KLF2) or control plasmids into HEK293T cells by Lipo2000 (11668027, Thermo Fisher). After transfection for 48 h, luciferase activity was detected following the instructions of Dual‐Lumi™ Luciferase Reporter Gene Assay Kit (Beyotime).

### 2.15 Chromatin immunoprecipitation (ChIP)

The ChIP experiment was performed according to the instructions of ChIP Assay kit (Beyotime). At room temperature, the cells were cross‐linked with 1% formaldehyde for 10 min and the cross‐link was terminated with 125 mM glycine. The DNA fragment was sonicated to 200–500 bp. ChIP was conducted using anti‐EZH2 (ab186006, 1:50, Abcam), anti‐H3K27me3 (ab192585, 1:50, Abcam) or rabbit anti‐IgG (ab172730, 1:50, Abcam). The immunoprecipitated DNA was analysed by RT‐qPCR. The primers of KLF2 were covered from the promoter to the upstream of the transcription start site of KLF2 gene. The sequences of KLF2 were as follows: Forward: 5′‐ACGGGCTTATTGAGGTTGG‐3′; Reverse: 5′‐GCCTGGGTGACAGAGGAGAC‐3′.

### 2.16 Tumour formation in vivo

A total of 40 BABL/c male nude mice (ageing 4–6 weeks, weighing 20.1 ± 3.6 g, Shanghai SLAC Laboratory Animal Co., Ltd.) were used in this experiment. Nude mice were kept at a humidity of 45%–50% with a temperature of 25–27°C, free access to food and water. A549, A549/lv‐NC and A549/lv‐miR‐126‐5p cells in logarithmic growth phase (the titre of the lentiviral vector was 2 × 10^9^ TU/ml, Genechem) were resuspended in 0.2 ml PBS. The cell concentration was adjusted to 1 × 10^7^ cells/ml. Cells were inoculated subcutaneously into the right posterior abdomen of nude mice. The tumour volume was measured every 3 days, and when the tumour volume reached 50 mm^3^, radiotherapy was conducted. Nude mice were randomly divided into four groups (*n* = 10): Control group (subcutaneous injection of A549 cells without radiotherapy), Radiotherapy group (subcutaneous injection of A549 cells and then underwent 20 Gy X‐ray radiation treatment), Radiotherapy +lv‐NC group (subcutaneous injection of A549/lv‐NC cells and then underwent 20 Gy X‐ray radiation treatment), Radiotherapy +lv‐miR‐126‐5p group (subcutaneous injection of A549/lv‐miR‐126‐5p cells and then underwent 20 Gy X‐ray radiation), radiotherapy was performed every 5 days and repeated three times. During this period, the volume and weight of the transplanted tumour were observed. On the 21st day after tumour cell inoculation, nude mice were killed, and tumour specimens were collected and stored at −80°C for RT‐qPCR and Western blot analysis.

### 2.17 Statistical analysis

Data analysis was performed using the GraphPad Prism 8.0. The measurement data are presented as mean ± standard deviation and analysed by unpaired *t*‐test or one‐way analysis of variance (ANOVA) with post hoc Tukey's test was used. The Kaplan‐Meier method was utilised to calculate the survival rate of patients. Values of *p* < .05 were considered significant.

## 3 RESULTS

### 3.1 miR‐126‐5p is poorly expressed in lung adenocarcinoma tissues and cells, and is associated with poor prognosis of patients with lung adenocarcinoma

**FIGURE 1:**
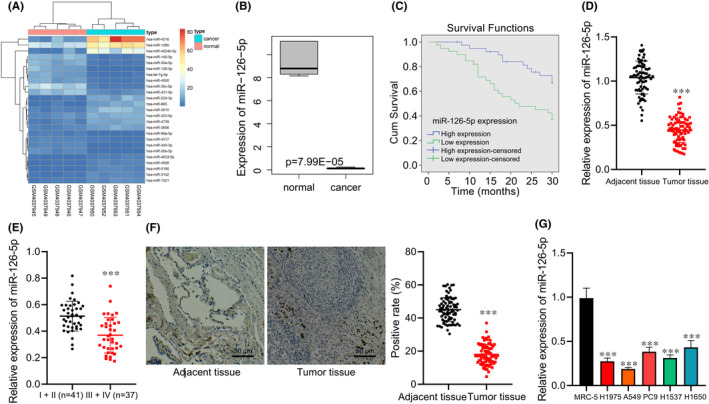
The expression of miR‐126‐5p in lung adenocarcinoma tissues and cells. (A) The expression heat map of the top 25 differentially expressed miRNAs of the lung adenocarcinoma‐related miRNA expression dataset GSE135918 (n = 5). (B) Box plot of miR‐126‐5p expression in the lung adenocarcinoma‐related dataset GSE135918 (*n* = 5). (C) Kaplan‐Meier curve analysis of the correlation between the miR‐126‐5p expression and prognosis of patients with lung adenocarcinoma (high expression, *n* = 30; low expression, *n* = 48). (D) miR‐126‐5p expression in lung adenocarcinoma tissues and adjacent normal tissues determined by RT‐qPCR (*n* = 78). (E) miR‐126‐5p expression in the early and advanced stage of patients with lung adenocarcinoma determined by RT‐qPCR (Ⅰ + Ⅱ = 41, Ⅲ + Ⅳ = 37). (F) miR‐126‐5p expression in lung adenocarcinoma tissues and adjacent normal tissues verified by ISH (*n* = 78, Scale bar = 50 μm). (G) miR‐126‐5p expression in five lung adenocarcinoma cell lines and human embryonic lung fibroblast cell lines determined by RT‐qPCR. Data are shown as the mean ± standard deviation of three technical replicates. Data comparisons between two groups were analysed by unpaired *t*‐test. Data comparisons among multiple groups were analysed by the one‐way ANOVA with Tukey's post hoc test. *p < 0.05; **p < 0.01; ***p < 0.001

The differential analysis of the miRNA expression dataset GSE135918 related to lung adenocarcinoma was performed, revealing that differential expression multiple of hsa‐miR‐145‐5p, hsa‐miR‐30c‐5p, hsa‐miR‐431‐5p and hsa‐miR‐126‐5p were the highest and significantly downregulated (Figure 1A). It has been reported that miR‐126‐5p was under‐expressed in NSCLC,^15^ which was consistent with the results of dataset GSE135918 (Figure 1B), but there are few studies on its effect on lung adenocarcinoma and its possible mechanism. Therefore, miR‐126‐5p was selected for the experiments.

The follow‐up period of enrolled patients was 2–30 months and the survival rate was analysed by Kaplan‐Meier method. We found that the overall survival of patients with lung adenocarcinoma with high expression of miR‐126‐5p was obviously longer than those with low expression of miR‐126‐5p (Figure 1C). Besides, lower miR‐126‐5p expression was seen in lung adenocarcinoma tissues (Figure 1D); and miR‐126‐5p expression in patients with lung adenocarcinoma at the advanced stage was lower than those at early stage (Figure 1E). ISH assay confirmed low miR‐126‐5p expression in the lung adenocarcinoma tissues (Figure 1F).

It was further verified in five lung adenocarcinoma cell lines and human embryonic lung fibroblast cell lines by RT‐qPCR that miR‐126‐5p expression in lung adenocarcinoma cells was obviously lower than that in human embryonic lung fibroblasts. Among the five lung adenocarcinoma cell lines, the miR‐126‐5p expression was relatively lower in the A549 cell line, and relatively higher in the H1650 cell line, and these two cell lines were selected for subsequent experiments (Figure 1G).

### 3.2 miR‐126‐5p enhances radiosensitivity

**FIGURE 2:**
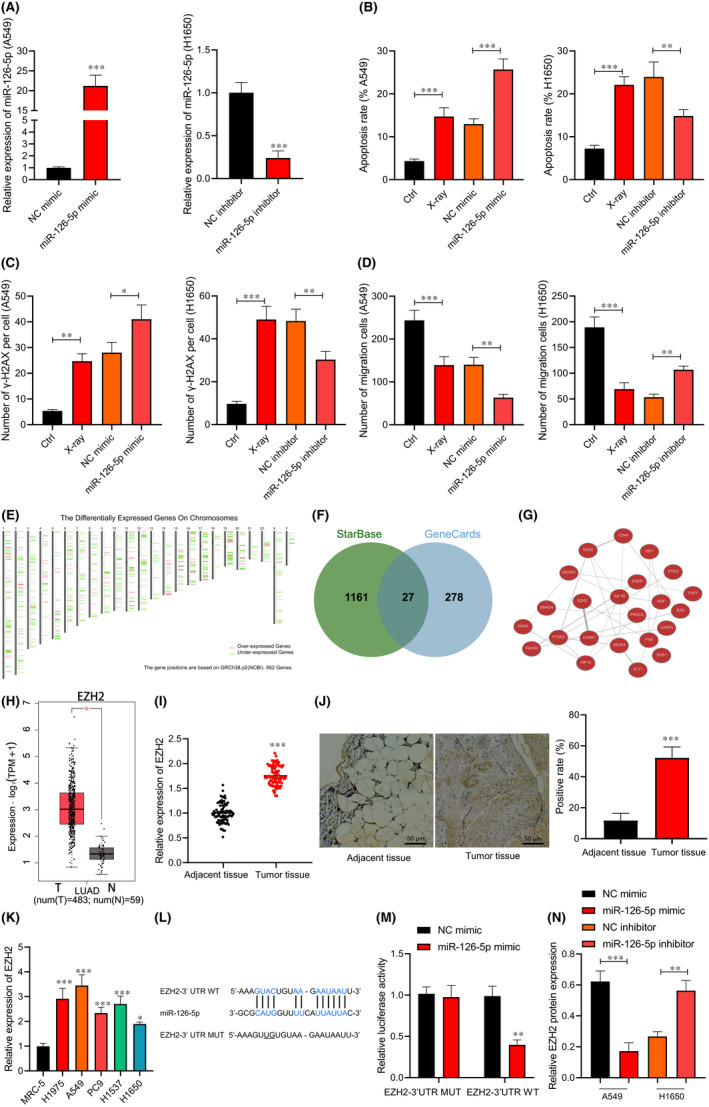
miR‐126‐5p expression affects the radiosensitivity of lung adenocarcinoma cells. A549 and H1650 cells were treated with 10 Gy of X‐ray and transduced with miR‐126‐5p mimic or inhibitor. (A) The overexpression and silencing efficiency of miR‐126‐5p detected by RT‐qPCR. (B) A549 and H1650 cell apoptosis detected by flow cytometry. (C) The number of γ‐H2AX focus in A549 and H1650 cells detected by immunofluorescence staining. (D) A549 and H1650 cell migration detected by Transwell assay. (E) The positions of significantly DEGs in lung adenocarcinoma on chromosomes obtained using GEPIA2 tool. (F) The Venn map of the intersection of prediction results in the StarBase and GeneCards. (G) The co‐expression relationship network diagram of the candidate target genes using the Coexpedia website. (H) Box plot of high expression of EZH2 in lung adenocarcinoma using the GEPIA2 tool. (I) EZH2 expression in lung adenocarcinoma tissues and adjacent normal tissues determined by RT‐qPCR (*n* = 78). (J) EZH2 expression in lung adenocarcinoma tissues and adjacent normal tissues verified by immunohistochemical staining (*n* = 78, Scale bar = 25 μm). (K) EZH2 expression in five lung adenocarcinoma cell lines and human embryonic lung fibroblast cell lines determined by RT‐qPCR. (L) The binding site of miR‐126‐5p and target gene EZH2 predicted by StarBase website. (M) The targeted binding of miR‐126‐5p to EZH2 verified using the dual‐luciferase reporter gene assay. The A549 and H1650 cells were transduced with miR‐126‐5p mimic or miR‐126‐5p inhibitor. (N) EZH2 protein level in A549 and H1650 cells measured by Western blot analysis. Data are shown as the mean ± standard deviation of three technical replicates. Data comparisons between two groups were analysed by unpaired *t*‐test. Data comparisons among multiple groups were analysed by the one‐way ANOVA with Tukey's post hoc test. *p < 0.05; **p < 0.01; ***p < 0.001

RT‐qPCR (Figure 2A) presented that after transfection for 24 h, the miR‐126‐5p expression increased by ~20 times in A549 cells, and decreased by ~80% in H1650 cells. Next, flow cytometry (Figure 2B), immunofluorescence staining (Figure 2C) and Transwell assay (Figure 2D) revealed that after X‐ray treatment, the apoptosis and γ‐H2AX focus number increased and migration of A549 and H1650 cells was inhibited. However, after X‐ray treatment, A549 cells transduced with miR‐126‐5p mimic showed promoted apoptosis, increased number of γ‐H2AX focus, and suppressed migration. However, opposite changing tendency was seen in H1650 cells transduced with miR‐126‐5p inhibitor after X‐ray treatment.

### 3.3 miR‐126‐5p targets and inhibits EZH2

In order to explore the downstream target genes regulated by miR‐126‐5p in lung adenocarcinoma, the differentially expressed genes in lung adenocarcinoma were obtained using the GEPIA2 tool (Figure 2E). A total of 1188 target genes of miR‐126‐5p were predicted by the StarBase and the lung adenocarcinoma‐related genes were obtained from the GeneCards database, and then 27 candidate target genes were collected after intersection (Figure 2F). The Coexpedia was used to obtain the co‐expression relationship network diagram of the candidate target genes (Figure 2G), in which EZH2 and CHEK1 had the highest scores on the website (Score >20). Existing literature indicated that EZH2 was upregulated in lung adenocarcinoma cells.^11^ Moreover, EZH2 was significantly highly expressed in lung adenocarcinomas through the GEPIA2 tool (Figure 2H). Thus, EZH2 was selected for the experiments.

Subsequently, we found higher EZH2 expression in lung adenocarcinoma tissues (Figure 2I,J). In addition, RT‐qPCR (Figure 2K) also confirmed elevated EZH2 in lung adenocarcinoma cells. Next, StarBase website was employed to predict the specific binding site between miR‐126‐5p and EZH2 (Figure 2L). Luciferase assay verified (Figure 2M) that luciferase activity of EZH2‐3’UTR‐WT was inhibited by miR‐126‐5p mimic (*p* < 0.05), while no evident difference was found in EZH2‐3’UTR‐MUT (*p* > 0.05), suggesting that EZH2 was a target gene of miR‐126‐5p. Western blot analysis (Figure 2N and Figure S1A) exhibited that EZH2 expression reduced in A549 cells transduced with miR‐126‐5p mimic, and it elevated in H1650 cells transduced with miR‐126‐5p inhibitor. Thus, miR‐126‐5p targeted and downregulated EZH2.

### 3.4 EZH2 inhibits KLF2 expression by inducing KLF2 H3K27me3 modification

**FIGURE 3:**
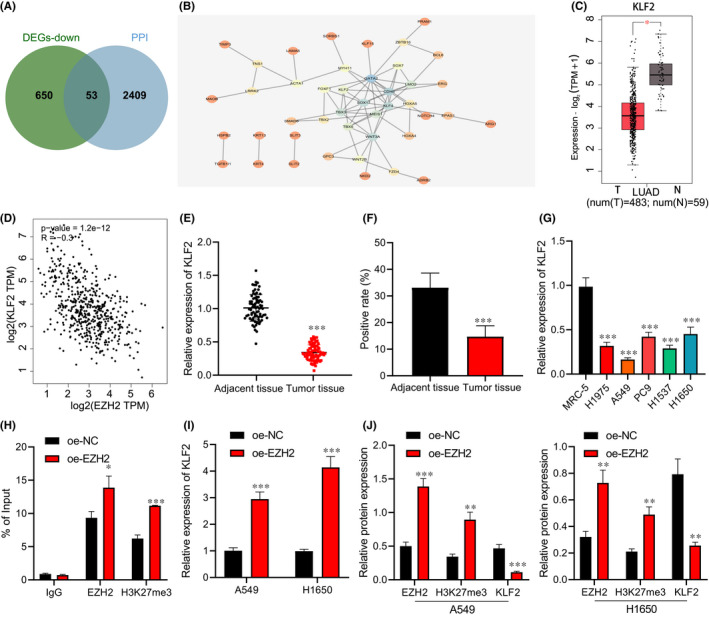
EZH2 affects KLF2 expression by inducing H3K27me3 modification. (A) Venn map of the intersection of the interacting protein of EZH2 and the significantly downregulated genes in the lung adenocarcinoma using the GeneCards database. (B) The interaction relationship between the significantly downregulated genes in the lung adenocarcinoma that interacted with EZH2. The colour scales from blue to orange indicate the degree value of the gene from large to small. (C) The box plot of KLF2 expression in lung adenocarcinoma samples. (D) Correlation between EZH2 and KLF2 in lung adenocarcinoma. (E) KLF2 expression in lung adenocarcinoma tissues and adjacent normal tissues determined by RT‐qPCR (*n* = 78). (F) KLF2 protein level in lung adenocarcinoma tissue and adjacent normal tissues determined by immunohistochemical staining (*n* = 78). (G) KLF2 expression in five lung adenocarcinoma cell lines and human embryonic lung fibroblast cell lines determined by RT‐qPCR. (H) The enrichment of EZH2 and H3K27me3 in the KLF2 promoter region detected by ChIP‐PCR. A549 and H1650 cells were transduced with oe‐EZH2. (I) The overexpression efficiency of EZH2 in A549 and H1650 cells detected by RT‐qPCR. (J) Protein levels of EZH2, H3K27me3 and KLF2 in A549 and H1650 cells measured by Western blot analysis. Data are shown as the mean ± standard deviation of three technical replicates. Data comparisons between two groups were analysed by unpaired *t*‐test. Data comparisons among multiple groups were analysed by the one‐way ANOVA with Tukey's post hoc test. *p < 0.05; **p < 0.01; ***p < 0.001

Next, 2462 interacting proteins of EZH2 were found in the GeneCards database, which were intersected with the significantly lowly‐expressed DEGs in lung adenocarcinoma, then 53 genes were collected (Figure 3A). In the interaction network diagram of 53 genes, 10 genes were at the core position (Degree ≥5; Figure 3B), among which KLF2 was obviously downregulated in lung adenocarcinoma tissues (Figure 3C), and there was a significant correlation between EZH2 and KLF2 in lung adenocarcinoma (Figure 3D). A prior study has suggested that EZH2 can be enriched in the KLF2 promoter region to inhibit KLF2 transcription, leading to the promotion the proliferation of NSCLC cells.^16^ Therefore, KLF2 was selected for the following experiments. RT‐qPCR and immunohistochemical staining (Figure 3E–G) exhibited that KLF2 expression decreased in lung adenocarcinoma tissues and cells (Figure 3F).

ChIP‐PCR (Figure 3H) showed that the EZH2 and H3K27me3 was enriched in the KLF2 gene promoter region in A549 and H1650 cells transduced with oe‐EZH2. Next, A549 and H1650 cells were transduced with oe‐EZH2, and we observed that the protein levels of EZH2 and H3K27me3 increased and KLF2 protein level decreased in A549 and H1650 cells transduced with oe‐EZH2 (Figure 3I,J and Figure S1B). It could be concluded that EZH2 could be enriched in the KLF2 promoter region to trigger H3K27me3 modification, thereby inhibiting the KLF2 expression.

### 3.5 Transcription factor KLF2 inhibits BIRC5 transcription

**FIGURE 4:**
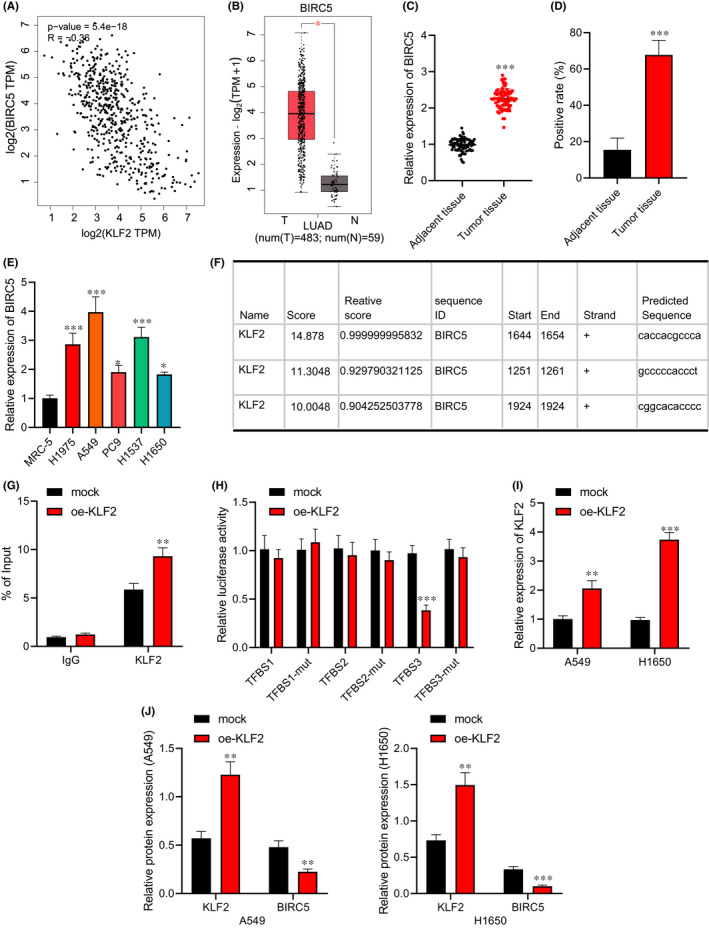
KLF2 affects BIRC5 expression by binding to the promoter region of BIRC5. (A) Correlation analysis of KLF2 and BIRC5 in lung adenocarcinoma. (B) Box plot of BIRC5 expression in lung adenocarcinoma tissue samples. (C) BIRC5 expression in lung adenocarcinoma tissues and adjacent normal tissues determined by RT‐qPCR (*n* = 78). (D) BIRC5 protein level in lung adenocarcinoma tissue and adjacent normal tissues determined by immunohistochemical staining (*n* = 78, Scale bar = 100 μm). (E) BIRC5 expression in five lung adenocarcinoma cell lines and human embryonic lung fibroblast cell lines determined by RT‐qPCR. (F) The possible binding site of KLF2 and BIRC5 promoter predicted by JASPAR website. (G) The degree of KLF2 enrichment in BIRC5 gene promoter region detected by ChIP‐PCR. (H) The binding site of KLF2 and BIRC5 promoter region verified using the dual‐luciferase reporter gene assay. A549 and H1650 cells were transduced with oe‐KLF2 after irradiation. (I) The transfection efficiency of KLF2 overexpression in A549 and H1650 cells detected by RT‐qPCR. (J) Protein levels of KLF2 and BIRC5 in A549 and H1650 cells measured by Western blot analysis. Data are shown as the mean ± standard deviation of three technical replicates. Data comparisons between two groups were analysed by unpaired *t*‐test. Data comparisons among multiple groups were analysed by the one‐way ANOVA with Tukey's post hoc test. *p < 0.05; **p < 0.01; ***p < 0.001

It was found that KLF2 and BIRC5 were negatively correlated in lung adenocarcinoma through GEPIA2 (Figure 4A), and BIRC5 was significantly highly expressed in lung adenocarcinoma (Figure 4B). It is known that BIRC5, a proto‐oncogene encoding survivin protein, can regulate the apoptosis and senescence of NSCLC cells, and its expression is significantly related to the survival rate of patients with lung adenocarcinoma.^17^ Therefore, we speculated whether KLF2 played a role in lung adenocarcinoma by regulating BIRC5 expression.

RT‐qPCR (Figure 4C,E) showed that BIRC5 expression increased in lung adenocarcinoma tissues and cells, and the same result was found by the immunohistochemical staining (Figure 4D). The JASPAR website predicted the possible binding site of KLF2 in the BIRC5 promoter (Figure 4F), and ChIP‐PCR (Figure 4G) displayed that the enrichment level of KLF2 in the BIRC5 gene promoter region of the KLF2 group was higher. Luciferase assay verified (Figure 4H) that luciferase activity of the plasmid transfected with KLF2‐3’UTR was inhibited by BIRC5 (*p* < 0.05), indicating that BIRC5 may act on the KLF2‐3’UTR promoter region.

Furthermore, A549 and H1650 cells were transduced with oe‐KLF2. RT‐qPCR (Figure 4I) exhibited that at 24 h after transfection, KLF2 expression increased in A549 and H1650 cells. Western blot analysis (Figure 4J and Figure S1C) presented that KLF2 expression elevated and BIRC5 expression reduced in the A549 and H1650 cells transduced with oe‐KLF2. The obtained data indicated that BIRC5 was a downstream target gene of KLF2, and negatively regulated by KLF2.

### 3.6 miR‐126‐5p enhances radiosensitivity by regulating the EZH2/KLF2/BIRC5 axis

**FIGURE 5:**
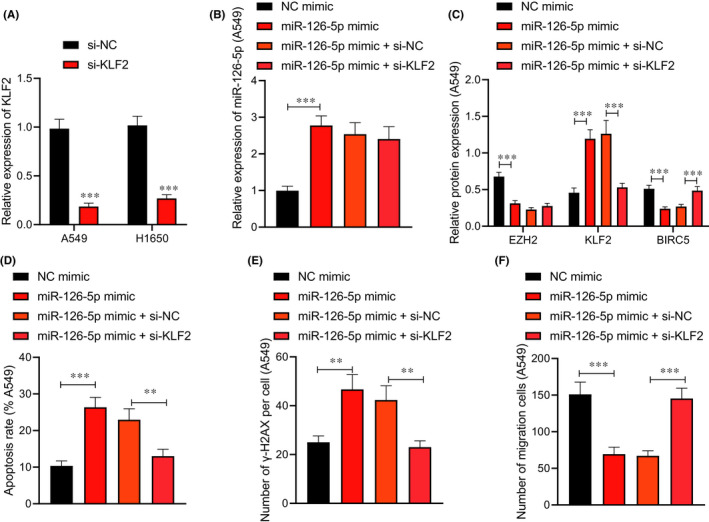
miR‐126‐5p affects the radiosensitivity of A549 cells via the EZH2/KLF2/BIRC5 axis. A549 cells were treated with X‐rays and transduced with miR‐126‐5p mimic alone or combined with si‐KLF2. (A) The transfection efficiency of si‐KLF2 in A549 cells detected by RT‐qPCR. (B) miR‐126‐5p expression in A549 cells determined by RT‐qPCR. (C) Protein levels of EZH2, KLF2 and BIRC5 in A549 cells measured by Western blot analysis. (D) A549 cell apoptosis detected by flow cytometry. (E) The number of γ‐H2AX focus in A549 cells detected by immunofluorescence staining. (F) A549 cell migration detected by Transwell assay. Data are shown as the mean ± standard deviation of three technical replicates. Data comparisons between two groups were analysed by unpaired *t*‐test. Data comparisons among multiple groups were analysed by the one‐way ANOVA with Tukey's post hoc test. *p < 0.05; **p < 0.01; ***p < 0.001

We then study the effect of miR‐126‐5p regulating the EZH2/KLF2/BIRC5 axis on the radiosensitivity of lung adenocarcinoma cells. RT‐qPCR (Figure 5A) displayed that at 24 h after transfection, KLF2 expression decreased in A549 and H1650 cells after si‐KLF2 treatment. Moreover, an increase in miR‐126‐5p and KLF2 expression, and a reduction in EZH2 and BIRC5 expression were found in A549 and H1650 cells transduced with miR‐126‐5p mimic, while expression of miR‐126‐5p and EZH2 showed no obvious difference, KLF2 expression reduced, and BIRC5 expression elevated in A549 and H1650 cells transduced with miR‐126‐5p mimic and si‐KLF2 (Figure 5B,C and Figure S2A,B).

Next, flow cytometry (Figure 5D and Figure S2C), immunofluorescence staining (Figure 5E and Figure S2D), and Transwell assay (Figure 5F and Figure S2E) showed that elevated miR‐126‐5p promoted A549 and H1650 cell apoptosis, increased γ‐H2AX focus number, and inhibited cell migration, while simultaneous elevated miR‐126‐5p and silencing KLF2 exerted the opposite effects on the A549 and H1650 cells. These findings proved that that miR‐126‐5p downregulated the EZH2 expression and increased the KLF2 expression to inhibit the BIRC5 activation, ultimately enhancing radiosensitivity.

### 3.7 miR‐126‐5p increases radiosensitivity via the EZH2/KLF2/BIRC5 axis in vivo

**FIGURE 6:**
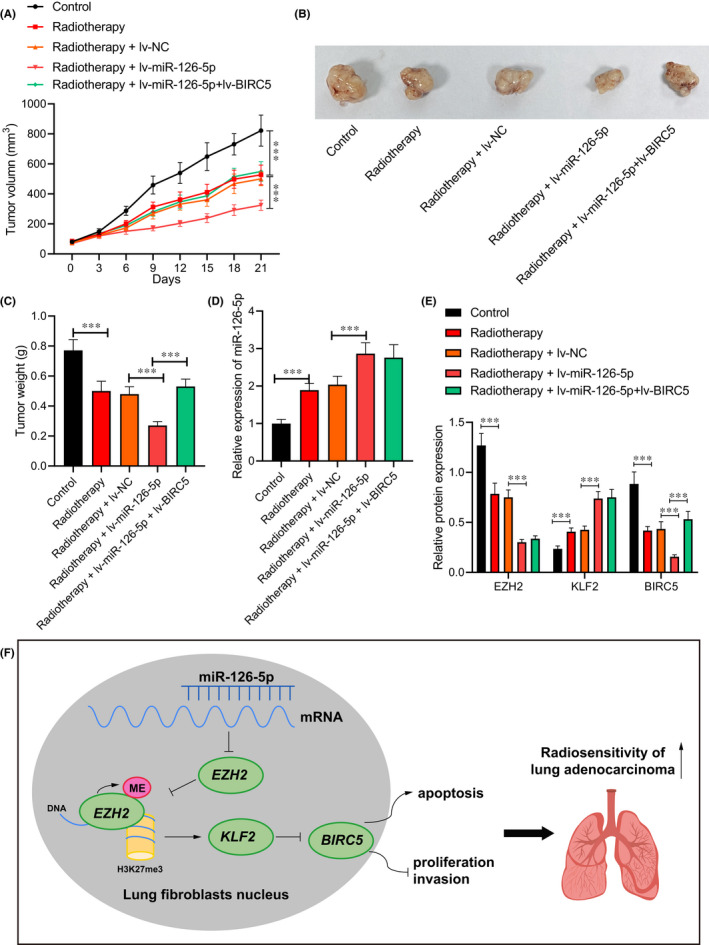
miR‐126‐5p affects the radiosensitivity of lung adenocarcinoma cells via the EZH2/KLF2/BIRC5 axis in vivo. Nude mice were treated with 20 Gy X‐ray alone or combined with lv‐miR‐126‐5p. (A) Tumour volume of nude mice. (B) Xenograft tumour in nude mice. (C) Tumour weight of nude mice. (D) miR‐126‐5p expression in tumour tissues of nude mice determined by RT‐qPCR. (E) Protein levels of EZH2, KLF2, and BIRC5 in tumour tissues of nude mice measured by Western blot analysis. (F) Molecular mechanism of miR‐126‐5p involved in regulating the EZH2/KLF2/BIRC5 axis to improve the radiosensitivity of lung adenocarcinoma cells. Data are shown as the mean ± standard deviation of three technical replicates. Data comparisons between two groups were analysed by unpaired *t*‐test. Data comparisons among multiple groups were analysed by the one‐way ANOVA with Tukey's post hoc test. *p < 0.05; **p < 0.01; ***p < 0.001

Finally, the above mechanism was analysed in vivo. It was found that the volume (Figure 6A) and weight (Figure 6B,C) of transplanted tumour in nude mice decreased in nude mice treated with X‐ray alone or combined with lv‐miR‐126‐5p. Additional lv‐BIRC5 increased the volume and weight of tumours in presence of lv‐miR‐126‐5p after radiotherapy. An enhancement of miR‐126‐5p and KLF2 while reductions in EZH2 and BIRC5 were seen in tumour tissues of nude mice treated with X‐ray alone or combined with lv‐miR‐126‐5p. However, miR‐126‐5p expression did not differ significantly following delivery of lv‐BIRC5 in presence of lv‐miR‐126‐5p after radiotherapy (Figure 6D,E). The obtained data indicated that miR‐126‐5p promoted radiosensitivity of lung adenocarcinoma cells via the EZH2/KLF2/BIRC5 axis while overexpressed BIRC5 reversed the action of miR‐126‐5p.

## 4 DISCUSSION

Although great achievement has made in treatment strategies of lung adenocarcinoma, the progressive tolerance of lung adenocarcinoma cells in radiotherapy often leads to local recurrence and poor prognosis.^18^ Thus, it is urgent for discovering new potential therapeutic targets for lung adenocarcinoma for increasing radiosensitivity. The objective of our study was to investigate the effect of miR‐126‐5p, EZH2, KLF2 and BIRC5 in radio‐resistance of lung adenocarcinoma cells and their inner mechanisms. Data obtained in our study demonstrated that miR‐126‐5p‐mediated EZH2 exerted suppressive effects on proliferation, invasion and inducing effect on lung adenocarcinoma cell radiosensitivity and apoptosis via interaction between KLF2 and BIRC5.

We identified low miR‐126‐5p expression in lung adenocarcinoma tissues and cells, which was associated with poor prognosis. It is reported that many miRNAs, emerged as promising biomarkers for cancer treatment, are involved in the progression of multiple cancers, and have become promising biomarkers.^5^ miRNAs are reported to be dysregulated in lung adenocarcinoma.^19^ Meanwhile, similar result was seen in previous study that miR‐126‐5p was downregulated in lung adenocarcinoma.^8^ Moreover, elevated miR‐126‐5p enhanced the radiosensitivity of lung adenocarcinoma cells. Growing evidence has demonstrated the important role of miRNAs in regulation of the radiosensitivity of cancer cells.^9^ Partly in line with our study, a recent study also demonstrated the promotive effect of miR‐126‐5p in the sensitivity of clinical DDP treatment of NSCLC through negatively regulating ADAM9.^20^

In addition, the findings in this study proved that miR‐126‐5p targeted and inhibited EZH2 expression to facilitate the radiosensitivity of lung adenocarcinoma cells. Similarly, EZH2 is identified as a direct target of miR‐126.^21^ Multiple evidences have suggested that EZH2 is aberrantly expressed in lung adenocarcinoma.^22^, ^23^ EZH2 is capable of inducing cell proliferation, invasion and migration so as to enhance cancer progression.^10^ Upregulated EZH2 promotes lung adenocarcinoma cell invasiveness and metastasis in contribution to the progression of lung adenocarcinoma.^11^ EZH2 plays a role in metastatic disease recurrence following radiotherapy.^12^ At the same time, it has been explored that EZH2 contributes to the radioresistant phenotype of lung adenocarcinoma cells, providing the novel insights for investigating clinical treatment targets.^24^ Moreover, the target relation between miR‐126 and EZH2 in chemosensitivity in gastric cancer cells has been proved previously.^21^ These findings support that miR‐126‐5p facilitated the radiosensitivity of lung adenocarcinoma cells by targeting EZH2.

Furthermore, this study also indicated that EZH2 inhibited KLF2 expression to repress the radiosensitivity of lung adenocarcinoma cells. As a catalytic component of the polycomb repressive complex 2, EZH2 is a histone methyltransferase to suppress the specific gene expression.^25^ A prior study has proved that EZH2 can inhibit KLF2.^26^ KLF2 is poorly expressed in various cancers and possesses tumour‐suppressive features by repressing cell proliferation.^27^ Downregulated KLF2 is proved to promote the progression of NSCLC by binding to EZH2.^28^ Additionally, KLF2 suppressed BIRC5 to promote the radiosensitivity of lung adenocarcinoma cells. BIRC5 has been demonstrated to act as an oncogene to modulate the growth, migration and invasion of various cancer cells.^29^ Silencing of BIRC5 inhibited cell proliferation and colony formation but enhanced apoptosis and radiosensitivity of lung adenocarcinoma cells.^30^ Therefore, it can be concluded that miR‐126‐5p facilitated proliferation, invasion and radiosensitivity of lung adenocarcinoma cells and repressed apoptosis by targeting EZH2 via interaction between KLF2 and BIRC5.

To sum up, our study demonstrated that miR‐126‐5p inhibited EZH2 to elevate KLF2 expression and reduced BIRC5 expression, all of which leads to the repression of cell proliferation, migration and enhancement of radiosensitivity of lung adenocarcinoma cells and apoptosis (Figure 6F). Our findings identify an effective therapeutic strategy against radio‐resistance of lung adenocarcinoma cells. Due to the limited known researches, the roles of miR‐126‐5p, EZH2, KLF2, BIRC5 as well as their interaction in the radio‐resistance of lung adenocarcinoma cells should be more clearly investigated.

## CONFLICT OF INTEREST

The authors declare that they have no competing interests.

## AUTHOR CONTRIBUTIONS

**Fushi Han:** Data curation (equal); Formal analysis (equal); Writing – review & editing (equal). **Dongdong Huang:** Investigation (equal); Supervision (equal). **Jinqian Meng:** Methodology (equal); Project administration (equal). **Jiapeng Chu:** Resources (equal); Software (equal). **Meng Wang:** Validation (equal); Writing – review & editing (equal). **Shuzhen Chen:** Conceptualization (equal); Writing – original draft (equal).

## Supporting information

Figure S1

Figure S2

Table S1–S2

## Data Availability

The data that support the findings of this study are available from the corresponding author upon reasonable request.
