# Supplementary figures and images for: miR‐126‐5p enhances radiosensitivity of lung adenocarcinoma cells by inhibiting EZH2 via the KLF2/BIRC axis
Source: J Cell Mol Med. 2022 Mar 24;26(9):2529–42. doi: 10.1111/jcmm.17135 (PMC9077299; doi:10.1111/jcmm.17135)

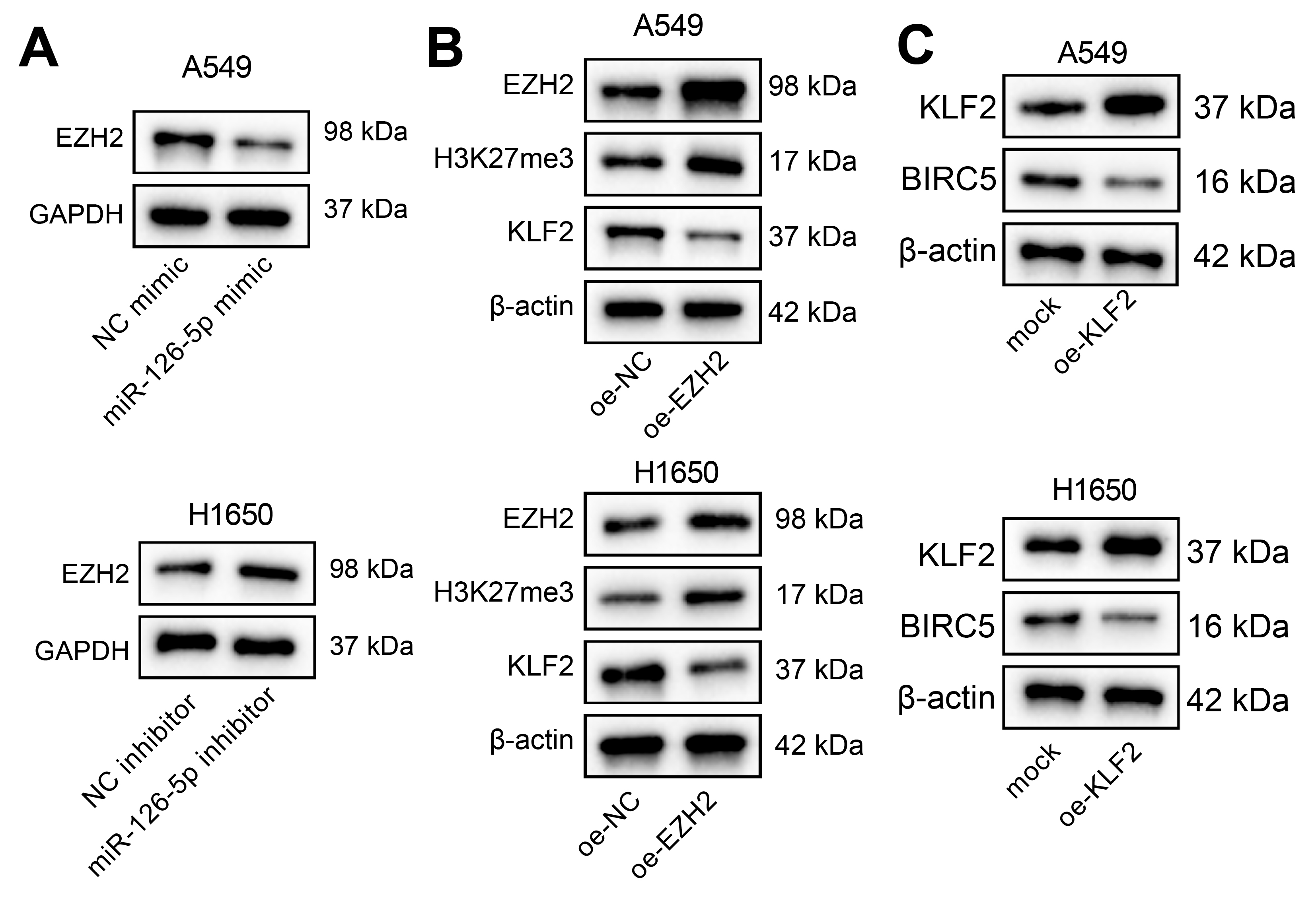

Supplement: Supplementary file 1 — Figure S1 [file JCMM-26-2529-s003.jpg]

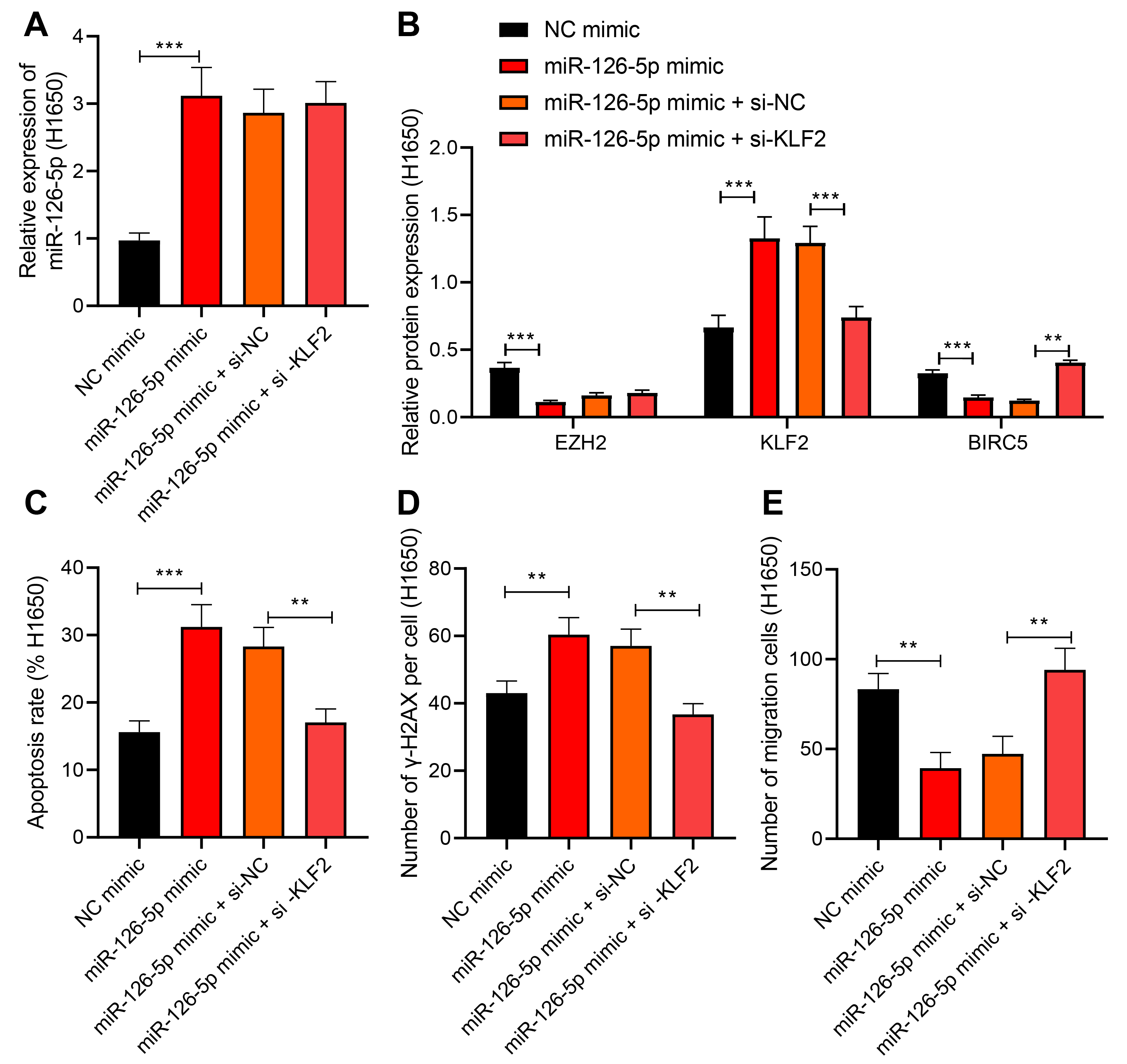

Supplement: Supplementary file 2 — Figure S2 [file JCMM-26-2529-s002.jpg]
