# Supplementary material for: miR‐126‐5p enhances radiosensitivity of lung adenocarcinoma cells by inhibiting EZH2 via the KLF2/BIRC axis
Source: J Cell Mol Med. 2022 Mar 24;26(9):2529–42. doi: 10.1111/jcmm.17135 (PMC9077299; doi:10.1111/jcmm.17135)
Supplement: Supplementary file 3 — Table S1–S2 [file JCMM-26-2529-s001.docx]

**Table S1** Clinical sample information of patients with lung adenocarcinoma

| Variable | Cases (n) | Percentage (%) |
| --- | --- | --- |
| Ages |  |  |
| < 60 | 63 | 80.77 |
| ≥ 60 | 15 | 19.23 |
| Gender |  |  |
| Male | 47 | 60.26 |
| Female | 31 | 39.74 |
| Clinical stage |  |  |
| I | 23 | 29.49 |
| II | 18 | 23.08 |
| III | 26 | 33.33 |
| IV | 11 | 14.10 |
| Tumor size |  |  |
| ≤ 3 cm | 57 | 73.08 |
| > 3 cm | 21 | 26.92 |
| Lymph node metastasis |  |  |
| No | 42 | 53.85 |
| Yes | 36 | 46.15 |
| Pleural invasion |  |  |
| No | 49 | 62.82 |
| Yes | 29 | 37.18 |

**Table S2** RT-qPCR primer sequences

| Genes | Primer (5′-3′) |
| --- | --- |
| GAPDH | Forward: GGAGCGAGATCCCTCCAAAAT |
|  | Reverse: GGCTGTTGTCATACTTCTCATGG |
| U6 | Forward: CTCGCTTCGGCAGCACA |
|  | Reverse: AACGCTTCACGAATTTGCGT |
| miR-126-5p | Forward: CGGCGCGTACCAAAAGT |
|  | Reverse: GTGCAGGGTCCGAGGT |
| EZH2 | Forward: TGCACATCCTGACTTCTGTG |
|  | Reverse: AAGGGCATTCACCAACTCC |
| KLF2 | Forward: CTGCACATGAAACGGCACAT |
|  | Reverse: CAGTCACAGTTTGGGAGGGG |
| BIRC5 | Forward: AGGACCACCGCATCTCTACAT |
|  | Reverse: AAGTCTGGCTCGTTCTCAGTG |

**Note:** RT-qPCR, reverse transcription quantitative polymerase chain reaction; GAPDH, glyceraldehyde-3-phosphate dehydrogenase; miR, microRNA; EZH2, enhancer of zeste homolog 2; KLF2, Kruppel like factor 2; BIRC5, baculoviral IAP repeat containing 5.
